# The complete chloroplast genome of *Eria lasiopetala* (Willd.) Ormerod

**DOI:** 10.1080/23802359.2021.1996293

**Published:** 2021-11-12

**Authors:** Yun Teng, Li Yang, Yan Zhang

**Affiliations:** College of Horticulture, Xinyang Agriculture and Forestry University, Xinyang, Henan, China

**Keywords:** *Dendrolirium lasiopetalum*, Orchidaceae, plastid genome, phylogenetic analysis

## Abstract

*Eria lasiopetala* (Willd.) Ormerod is an important ornamental plant and traditional Chinese medicine resource, but it is currently listed as a class II protected species due to its few resources in China. To further clarify the taxonomic status as well as provide genetic information for its conservation, the chloroplast genome of *E. lasiopetala* was assembled and characterized in this study. Results show that the genome is 158,740 bp in length, including a large single copy, two inverted repeats, and a small single copy, their lengths are 88,062, 26,213, and 18,252 bp, respectively. There are 132 genes in the chloroplast genome, among which the number of encoding protein genes, rRNA genes, and tRNA genes are 86, 8, and 38, respectively. The phylogenetic tree clearly shows that *E. lasiopetala* and *Eria corneri* cluster together with 100% support, rather than with *Dendrobium* group.

*Eria lasiopetala* (Willd.) Ormerod also known as *Dendrolirium lasiopetalum* (Willdenow) S. C. Chen & J. J. Wood (Ormerod 1995; Chen and Wood 2005), is an epiphytic orchid found on trunks or rocks at an altitude of 1200–1700 m in Hainan, Guangdong, and Guangxi provinces of South China. Its inflorescence rachis, pedicels, and ovaries, the abaxial surface of bracts and sepals are densely covered with white tomentose. It is an important ornamental plant and traditional Chinese medicine resource but is currently listed as a class II protected species due to its few resources in China. So far, the classification of *E. lasiopetala* is inconsistent even at the genus level, so the two scientific names have also been accepted by different taxonomic databases, like in Kewscience (https://wcsp.science.kew.org/qsearch.do;jsessionid=9AC05A8B8125B51190E95753EF0DF8AB.kppapp05-wcsp), and in World Flora Online (http://www.worldfloraonline.org/taxon/wfo-0000917564), whose exact name is still uncertain. Therefore, to further clarify the taxonomic status as well as provide genetic information for its conservation, its chloroplast genome was assembled and characterized in this study.

Fresh leaf samples were collected in the Jianfengling mountain of Ledong County, Hainan Province, China (108.91 E, 18.72 N). The voucher specimen (2020JuEl02) was deposited in the herbarium of Xinyang Agriculture and Forestry University. Leaf genomic DNA was extracted using the CTAB method (Stefanova et al. 2013). The integrity of DNA was detected by agarose gel electrophoresis, and the purity of DNA was detected by using spectrophotometer Nanodrop 2000 UV-vis (Thermo Scientific, France). After the DNA quality meets sequencing requirements, a paired-end (PE) library (insert size with 400-bp) was constructed using the Illumina PE DNA library kit according to the manufacturer’s instructions and sequenced with a read length of PE150 on Illumina Hiseq X Ten at Beijing Novogene Co., Ltd. NGS QC Toolkit v2.3.3 was used to filter the raw data under the default settings (Patel and Jain 2012), and the adapters and low-quality bases in reads were removed to obtain the clean reads. With reference to the chloroplast genome of *Pholidota imbricata* (NC_048955), the chloroplast genome of *E. lasiopetala* was assembled from the clean reads using MIRA v4.0 and MITObim v1.9 software (Hahn et al. 2013), and then corrected by contigs assembled using SOAPdenovo v2.04 (Luo et al. 2012). Then, the genome was annotated using online software PGA with default parameters (Qu et al. 2019) and finally adjusted manually. tRNAs were predicted using tRNAscan-SE v2.0 (http://lowelab.ucsc.edu/tRNAscan-SE/) (Lowe and Eddy 1997) and ARAGORN v1.2.38 (Laslett and Canback 2004). GC values of the chloroplast genome were calculated by using Geneious v11.1.2 (Kearse et al. 2012). The maximum likelihood phylogenetic tree was constructed based on the chloroplast genomes of 31 species downloaded from NCBI database and the chloroplast genome of *E. lasiopetala*, MAFFT v1.4.0 (Biomatters Ltd., Auckland, New Zealand) plugin in Geneious v11.1.2 was used for multiple alignments of homologous protein-coding sequences (CDSs) extracted from all genomes, which were imported into MEGA v6 (Tamura et al. 2013), the chloroplast genome of *Oxalis corniculata* (Chen et al. 2021) was taken as the outgroup, the bootstrap value was set to 1000.

The chloroplast genome size of *E. lasiopetala* is 158,740 bp in length with a mean coverage of 201.8×, the GC content was 36.99%. It has a typical tetrad structure, including a large single copy (LSC), two inverted repeats (IR), and a small single copy (SSC), their lengths are 88,062, 26,213, and 18,252 bp, respectively. There are differences in GC values of LSC, IR, and SSC regions, the GC value of the IR region is the highest (43.2%), followed by LSC (34.7%), and the GC value of the SSC region is the lowest (30.3%).

There are 132 genes in the chloroplast genome of *E. lasiopetala*, among which the number of protein-coding genes, rRNA genes, and tRNA genes were 86, 8, and 38, respectively. In tRNA, *tRNA-GUG*, *tRNA-CAU*, *tRNA-CAA*, *tRNA-GAC*, *tRNA-GAU*, *tRNA-UGC*, *tRNA-ACG*, and *tRNA-GUU* have two copies for each. In addition, the four ribosomal RNAs have two copies for each, located in the reverse repeat region IRA and IRB, respectively. Among the ribosomal protein subunit coding genes, *rps19*, *rpl2*, *rpl23*, *ycf2*, *ndhB*, and *rps7* have two copies, and the rest have one copy. Most of the chloroplast genes do not have introns, only some genes have one or two introns. Among them, *tRNA-UUU*, *tRNA-UCC*, *tRNA-UAA*, *tRNA-GAU*, *tRNA-UGC*, *rps16*, *atpF*, *rpoC1*, *psbH*, *petD*, *rpl16*, *rpl2*, *ndhB*, and *ndhA* have one intron, while *ycf3* and *clpP* have two introns.

Phylogenetic analysis was performed on the homologous CDS sequences of 31 Orchidaceae species, the support of clustering is high (Figure 1). The phylogenetic tree shows that the genus *Eria* is the sister to the clade of *Phalaenopsis* and *Gastrochilus*, while *Dendrobium* is the sister to the genus *Liparis*. *Eria* and *Dendrobium* are nested in two sister clades. This result indicates that the species in this study should be classified as *Eria* species, and *E. lasiopetala* is a more accurate name than *D. lasiopetalum* for it. This result enriches Orchidaceae plants genetic information, also lays a foundation for the resources conservation, molecular breeding, and other chloroplast genetic engineering studies of *E. lasiopetala*.

**Figure 1.**
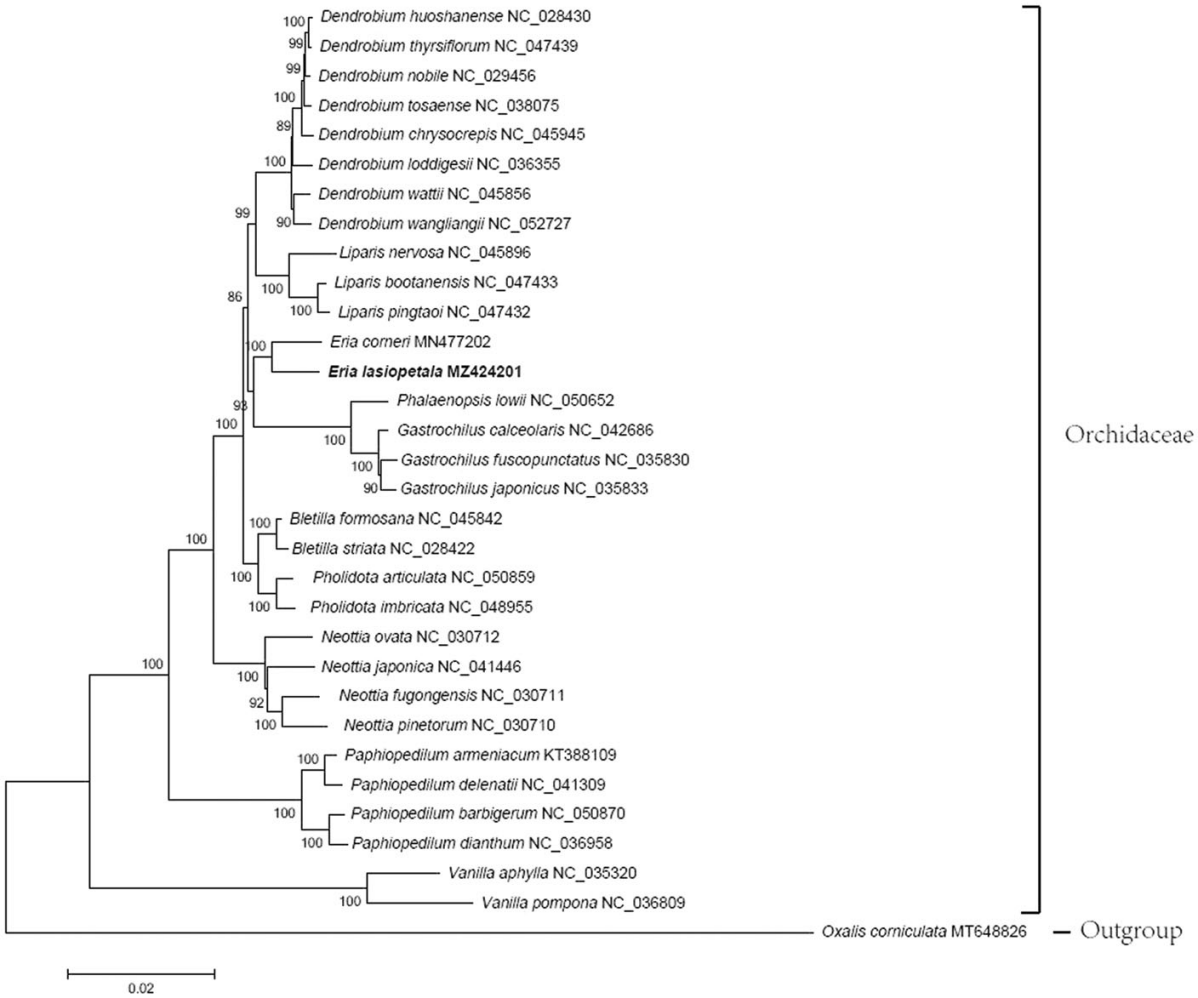
Phylogenetic relationships of Orchidaceae species inferred based on homologous protein-coding sequences of 31 chloroplast genomes. The accession numbers represent the chloroplast genomes deposited in the NCBI.

## Data Availability

The data that support the findings of this study are openly available in the National Genomics Data Center (NGDC), China National Center for Bioinformation. The raw reads and the chloroplast genome have been deposited under accession numbers of CRA004167 at https://bigd.big.ac.cn/gsa/browse/CRA004167 and GWHBAZG01000000 at https://bigd.big.ac.cn/search/?dbId=gwh&q=GWHBAZG01000000, respectively. The genome sequence is also available in GenBank of NCBI under accession no. MZ4242010, and the associated BioProject, SRA, and Bio-Sample numbers in NCBI are PRJNA740048, SRX11200455, and SAMN19817387, respectively.
